# Development of a novel ex vivo organ culture system to improve preservation methods of regenerative tissues

**DOI:** 10.1038/s41598-023-29629-2

**Published:** 2023-02-27

**Authors:** Tomomi Yuta, Tian Tian, Yuta Chiba, Kanako Miyazaki, Keita Funada, Kanji Mizuta, Yao Fu, Jumpei Kawahara, Tsutomu Iwamoto, Ichiro Takahashi, Satoshi Fukumoto, Keigo Yoshizaki

**Affiliations:** 1 Section of Orthodontics and Dentofacial Orthopedics, Division of Oral Health, Growth and Development, Kyushu University Faculty of Dental Science, Fukuoka, Japan; 2 Dento-Craniofacial Development and Regeneration Research Center, Kyushu University Faculty of Dental Science, Fukuoka, Japan; 3 Section of Pediatric Dentistry, Division of Oral Health, Growth and Development, Kyushu University Faculty of Dental Science, Fukuoka, Japan; 4 Department of Pediatric Dentistry/Special Needs Dentistry, Division of Oral Health Sciences, Graduate School of Medical and Dental Sciences, Tokyo Medical and Dental University, Tokyo, Japan; 5 Division of Pediatric Dentistry, Department of Community Social Dentistry, Tohoku University Graduate School of Dentistry, Sendai, Japan

**Keywords:** Developmental biology, Organogenesis

## Abstract

Recent advances in regenerative technology have made the regeneration of various organs using pluripotent stem cells possible. However, a simpler screening method for evaluating regenerated organs is required to apply this technology to clinical regenerative medicine in the future. We have developed a simple evaluation method using a mouse tooth germ culture model of organs formed by epithelial–mesenchymal interactions. In this study, we successfully established a simple method that controls tissue development in a temperature-dependent manner using a mouse tooth germ ex vivo culture model. We observed that the development of the cultured tooth germ could be delayed by low-temperature culture and resumed by the subsequent culture at 37 °C. Furthermore, the optimal temperature for the long-term preservation of tooth germ was 25 °C, a subnormothermic temperature that maintains the expression of stem cell markers. We also found that subnormothermic temperature induces the expression of cold shock proteins, such as cold-inducible RNA-binding protein, RNA-binding motif protein 3, and serine and arginine rich splicing factor 5. This study provides a simple screening method to help establish the development of regenerative tissue technology using a tooth organ culture model. Our findings may be potentially useful for making advances in the field of regenerative medicine.

## Introduction

The development of regenerative medicine is remarkable, and active research is being conducted to replace damaged organs with regenerative ones^1^. Researchers have already succeeded in regenerating organs, such as the liver^2,3^, heart^4,5^, and hair^6^ using human pluripotent stem cells and tissue engineering. However, regenerated organs take considerably longer to grow to a size and reach the stage of development appropriate for transplantation. Furthermore, even if regenerated organs could be grown, preservation methods for such organs have not yet been established. To ensure a stable supply of regenerated organs for patients, developing a technology to preserve pre-generated organs in conditions suitable for transplantation is necessary.

To date, various studies have been conducted to extend the tissue preservation time and improve the success rate of organ transplantation. Techniques include cold storage using various organ preservation solutions^7,8^, mechanical perfusion systems^9–11^, and cryopreservation methods^12^. Among these approaches, temperature control is a common factor that affects the duration of preservation. Cryopreservation is a well-developed method for the long-term preservation of cells. The first successful cryopreservation was that of sperm with glycerol as a cryoprotectant in 1949^13^. Cell cryopreservation technology for reproductive cells such as sperm^14^ and eggs^15,16^, blood^17,18^, and dental pulp stem cells^19^ has been applied clinically. In addition, the cryopreservation of cells derived from human pluripotent stem cells has been reported^20,21^. Cryopreservation still has several obstacles, such as intra-cellular ice formation^22^, cytotoxicity of the cryoprotectant^23^, and the inability of the cryoprotectant to penetrate all thick samples, such as large tissues or whole organs^24^. However, whole organ cryopreservation of ovarian^25,26^, liver^27^ and heart^28^ has been reported recently. Additionally, vitrification, which is an alternative to cryopreservation, is a method that solidifies water without ice formation to prevent cell damage induced by ice; therefore, it requires a high cooling and warming speed^22^. Organ preservation of heart^29^ and corneas^30^ via vitrification has also been reported. In contrast, although several studies have focused on methods for preserving tissues for transplantation, only few reports have focused on regenerating organs and developing organ preservation methods.

We previously established ex vivo culture methods for the tooth germ to screen various factors that affect developing organs^31–35^. Tooth development involves interaction between the ectoderm and neural crest-derived mesenchyme^36,37^. In mice, tooth development is initiated by the thickening of the dental epithelium and its invagination into the mesenchyme at embryonic day (E) 11.5. After epithelial invagination into the mesenchyme, the tooth germ forms a bud structure at E13.5 and a cap structure at E14.5^37^. Following the formation of a cusp structure, dental epithelial stem cells differentiate into ameloblasts and secrete enamel matrix proteins^38,39^. As the changes in morphological shape and gene expression are dynamic during tooth development, the degree of development can be easily assessed in ex vivo cultures. Furthermore, we can examine the direct effect of environmental factors, such as different temperatures, on organ conditions using this ex vivo culture method.

In this study, we analyzed the effect of temperature on tissue development using the mouse tooth germ ex vivo culture method. This study aimed to develop a method to validate the optimal temperature for the long-term preservation of tissue. For this purpose, we investigated the temperature conditions necessary for tissue maintenance, using mouse tooth germs which do not require the presence of blood vessels, to examine the effects of temperature changes on tooth germ constituent cells and organogenesis.

## Results

### Establishment of tissue preservation screening model using tooth germ ex vivo culture system

**Figure 1:**
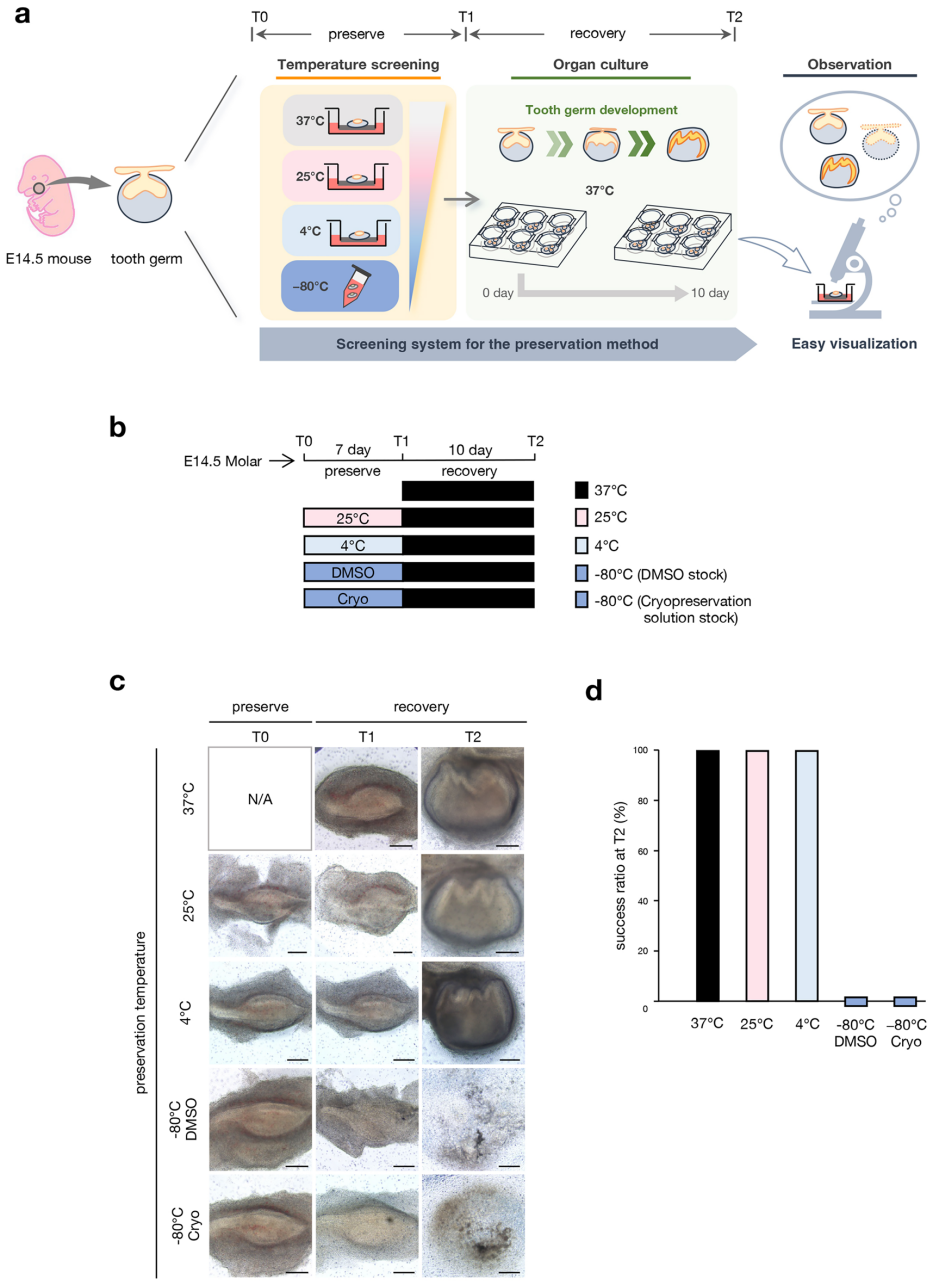
Comparison of tissue preservation conditions using ex vivo developing mouse tooth germ culture. (**a**) Schematic of an experimental procedure for the screening of tissue preservation conditions using E14.5 tooth germ organ culture. T0: preservation temperature starting point, T1: preservation temperature endpoint, T2: recovery temperature endpoint. (**b**) Time course representation of E14.5 tooth germs preserved at 37 °C control, at 25 °C, at 4 °C, in DMSO at − 80 °C, and in cryopreservation solution at − 80 °C for 7 days, followed by a recovery period at 37 °C for 10 days. (**c**) Photographic analysis of cultured E14.5 tooth germs under different temperature conditions in (**b**). Scale bars, 200 µm. (**d**) Analysis of the development rate of cultured tooth germs under different temperature conditions in (**c**) (n = 6).

We examined tissue preservation conditions using a tooth germ ex vivo culture system (Fig. 1a). We dissected tooth germs from E14.5 mouse embryos and cultured these at an air–liquid interface. An E14.5 tooth germ is histologically defined as the cap stage, and the morphological changes of the tooth germ are significant during ex vivo culture. Therefore, the developmental stage and survival of cultured tooth germs can be easily determined from their morphology. We hypothesized that organ damage and subsequent recovery depended on temperature and preservation conditions. We examined the preservation conditions by preserving the tooth germ at various temperatures (T0: preservation start point to T1: preservation endpoint) and subsequently placed it at 37 °C for 10 days for recovery (T1 to T2: recovery endpoint). Then, we observed the developmental stage of the tooth germ (Fig. 1a). In this study, the presence of cusp formation was defined as successful organogenesis, in order to use simple criteria. The dissected tooth germs were preserved for 7 days using four different methods: subnormothermic temperature preservation at 25 °C, cold-temperature preservation at 4 °C, cryopreservation in a tube filled with DMSO at − 80 °C, and cryopreservation with cryopreservation solution at − 80 °C (Fig. 1b). After 7 days, the preserved tooth germs were cultured at 37 °C for 10 days for recovery. The tooth germs cultured at 37 °C for 10 days were used as control (Fig. 1c, upper panel). Although the tooth germs preserved at − 80 °C did not survive past the recovery period, those preserved at 25 and 4 °C resumed development after removing them from the preservation conditions and showed cusp formation (Fig. 1c,d). We did not observe any differences in the developmental stage of the tooth germ between the 25 and 4 °C cultures (Supplemental Fig. S1). These results suggest that the tooth germs preserved at subnormothermic (25 °C) or cold temperature (4 °C) can resume development after the recovery period.

### Subnormothermic temperature can preserve tooth germ for longer than cold temperature

**Figure 2:**
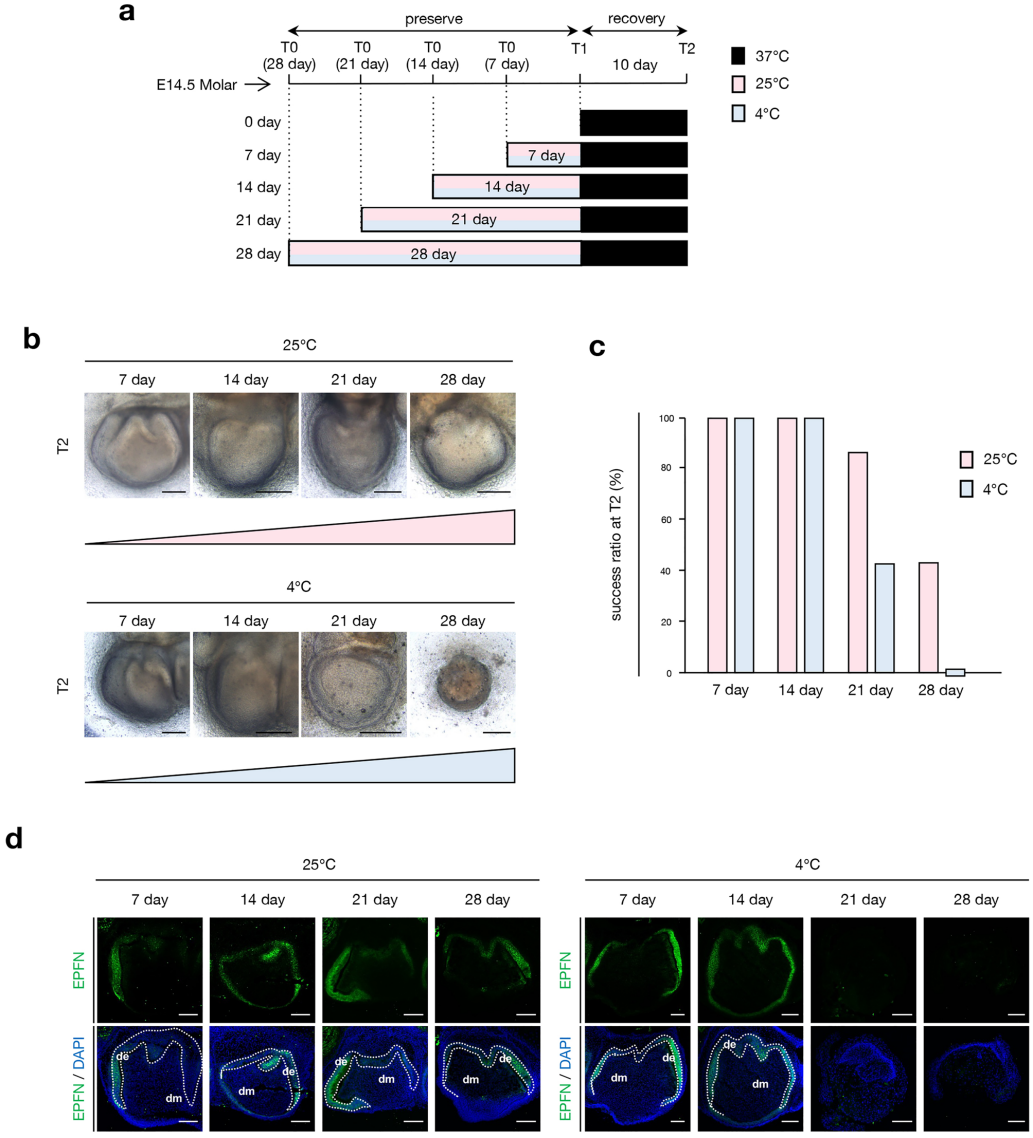
Comparison of low-temperature preservation conditions in extended-term preservation period. (**a**) Time course representation of tissue preservation for extended-term preservation at 37 °C control, low-temperature preservation (25 °C or 4 °C) for 7, 14, 21, and 28 days followed by recovery at 37 °C for 10 days. T0: preservation temperature starting point, T1: preservation temperature endpoint, T2: recovery temperature endpoint. (**b**) Photographic analysis of cultured E14.5 tooth germs in low-temperature preservation conditions for an extended term represented in (**a**). Scale bars, 200 µm. (**c**) Analysis of the development rate of cultured tooth germs under different temperature conditions and preservation terms in (**b**) (n = 7). (**d**) Immunofluorescence of cultured E14.5 tooth germs in low-temperature preservation conditions at T2 represented in (**b**). Green: EPFN, blue: DAPI. Dashed lines represent the border of the dental epithelium and mesenchyme. *de* dental epithelium, *dm* dental mesenchyme. Scale bars, 100 µm.

We examined whether extended preservation periods affected tooth development during the recovery period. Preserved tooth germs at 25 or 4 °C were cultured for 7, 14, 21, and 28 days as a preservation period (Fig. 2a). After each preservation period, tooth germs recovered at 37 °C for 10 days. We found that the success ratio of tooth germ development at 4 °C decreased after a longer period of preservation, i.e., over 21 days (Fig. 2b,c). Furthermore, the success ratio of preservation at 25 °C remained 40%, whereas that at 4 °C was 0% after 28 days of culture (Fig. 2c). We then evaluated the development of tooth germs histologically, with immunofluorescence of cultured tooth germs using an anti-epiprofin (EPFN) antibody (Fig. 2d). EPFN is expressed in differentiated ameloblasts during tooth development and used as an ameloblast marker. Tooth germs preserved at 25 °C expressed EPFN in ameloblasts after their recovery period (Fig. 2d, left panel), indicating that tooth development and ameloblast differentiation were resumed after preservation. However, tooth germs preserved at 4 °C for more than 21 days did not show EPFN expression (Fig. 2d, right panel). These results suggest that prolonged preservation at cold temperatures disturbs the recovery of the tooth germ. Furthermore, subnormothermic preservation can preserve the tooth germ longer than cold temperature preservation.

### Temperature-dependent preservation is beneficial for tissues other than the tooth germ

**Figure 3:**
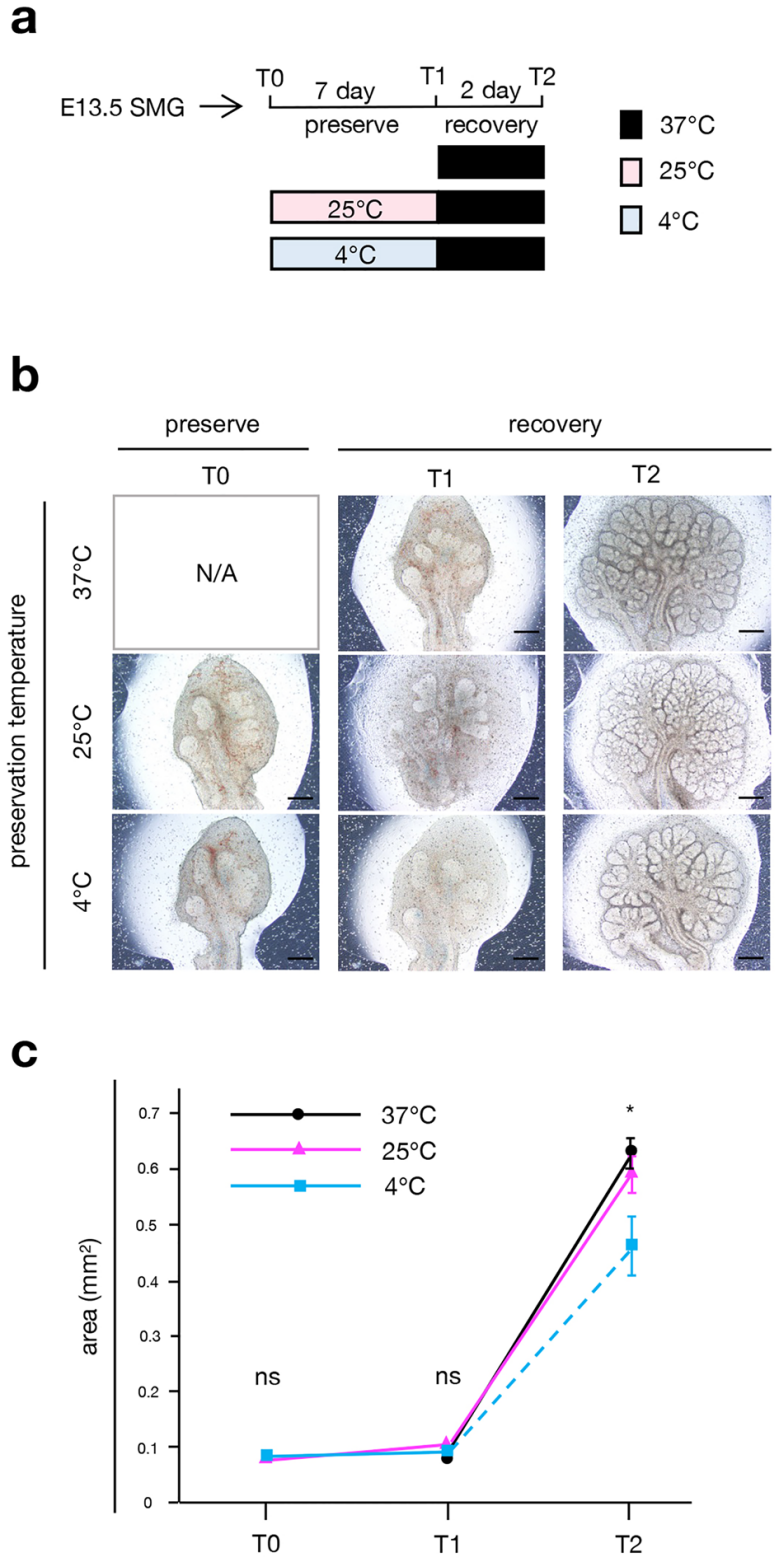
Comparison of tissue preservation condition using ex vivo developing submandibular gland culture. (**a**) Time course representation of tissue preservation at 37, 25, and 4 °C for 7 days, followed by a recovery period at 37 °C for 2 days. SMG: submandibular glands, T0: preservation temperature starting point, T1: preservation temperature endpoint, T2: recovery temperature endpoint. (**b**) Photographic analysis of cultured E13.5 submandibular glands in low-temperature preservation conditions represented in (**a**). Scale bars, 200 µm. (**c**) Changes in the area of cultured E13.5 submandibular gland buds at 37, 25, or 4 °C (n = 7). ns, p > 0.05: *p < 0.05. Error bars represent the mean ± SD.

To assess whether this temperature-dependent preservation can be used for tissues other than the tooth germ, we evaluated the preservation of E13.5 mouse submandibular glands following the same method (Fig. 3a). Dissected E13.5 submandibular glands were preserved at 25 or 4 °C for 7 days, and then cultured at 37 °C for 2 days (Fig. 3a). The submandibular glands branched and formed buds by organ culture method at 37 °C (Fig. 3b, upper panel). We observed that submandibular glands survived after 25 or 4 °C preservation, similar to tooth germ preservation (Fig. 3b, middle and lower panel). To study the differences between subnormothermic and cold-temperature preservation, we quantified the area of the glands post-recovery (Fig. 3c). The area of glands was not altered post-preservation at 25 °C. However, the area of the glands after cold-temperature preservation was significantly reduced compared to that in the control and subnormothermic preservation, suggesting that preservation at 4 °C disturbed the development of submandibular glands post-recovery.

### Low temperature delays the morphogenesis of cultured tooth germs

**Figure 4:**
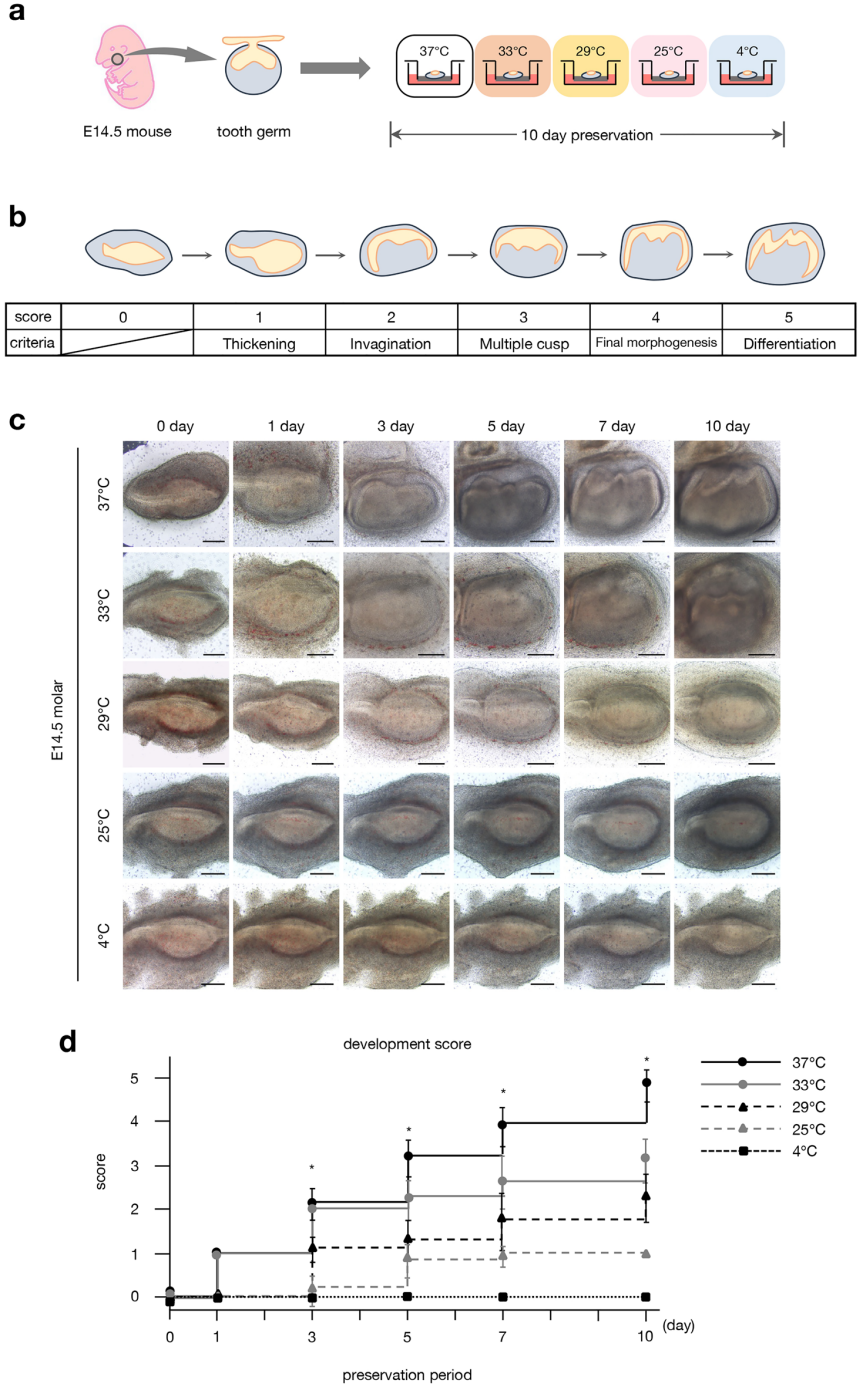
Low-temperature preservation arrests the morphogenesis of cultured tooth germs. (**a**) Schematic representation of an experimental procedure for comparing preservation conditions with different temperatures using an E14.5 tooth germ organ culture. (**b**) Schematic diagram of the assessment of morphological changes during tooth germ organ culture. The development of cultured tooth germs was scored 0 to 5 according to their morphological changes. Score 0: no apparent change from starting point; score 1: epithelium thickening; score 2: epithelial invagination into the mesenchyme; score 3: multiple cusp formation; score 4: final morphogenesis; score 5: differentiation. (**c**) Photographic analysis of cultured E14.5 tooth germs in different temperature preservation conditions for 0, 1, 3, 5, 7, and 10 days. Scale bars, 200 µm. (**d**) Analysis of the developmental score of cultured tooth germs at different temperatures and under different preservation conditions in (**c**) (n = 20). *p < 0.05. Error bars represent mean ± SD.

We further examined the effects of temperature-dependent preservation on tissue development using an ex vivo tooth germ culture system. To examine the effect of temperature conditions on tooth germ morphogenesis, E14.5 tooth germs were dissected and cultured at 37, 33, 29, 25, and 4 °C for 10 days (Fig. 4a). The process of cultured tooth morphogenesis was schematized, and the development processes were classified into five scores for evaluation: score 0, no noticeable change from the starting point; score 1, epithelium thickening; score 2, epithelial invagination into the mesenchyme; score 3, multiple cusp formation; score 4, final morphogenesis; score 5, differentiation (Fig. 4b). We examined the morphological changes in tooth germs cultured for 10 days and quantified the development score (Fig. 4c,d). We observed that a decline in temperature delayed the development of tooth germs. Notably, tooth germs cultured at 25 °C for 10 days exhibited a similar development score to those cultured at 37 °C for 1 day (Fig. 4c). Furthermore, tooth germs cultured at 4 °C exhibited no morphological changes during a 10-day culture period. These results suggest that low temperature can delay tooth morphogenesis.

### Subnormothermic temperature arrests changes in the expression of cellular differentiation marker genes and maintains the expression of stem cell marker genes

**Figure 5:**
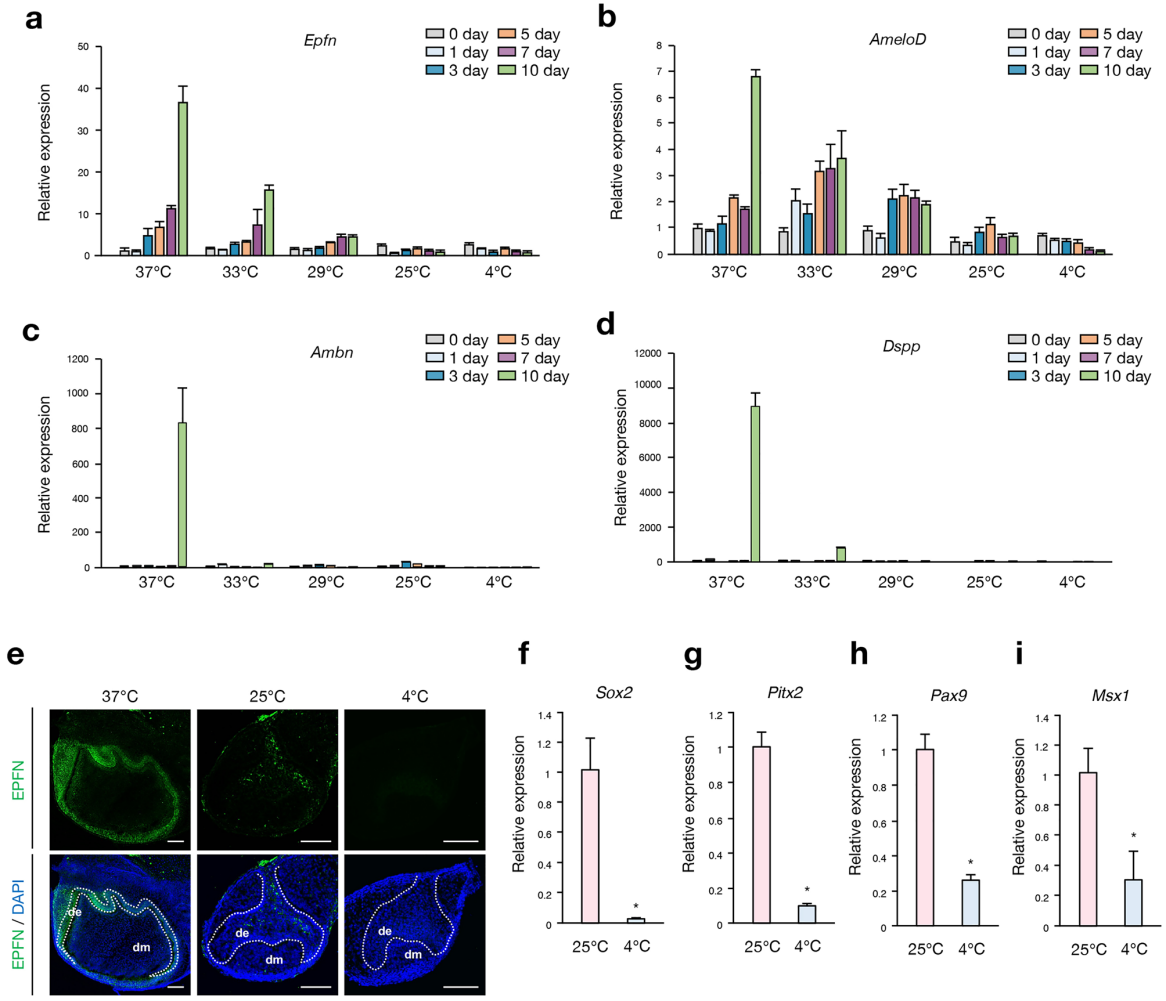
Subnormothermic temperature preservation maintains the expression of differentiation marker genes and stem cell marker genes. (**a**–**d**) RT-qPCR analysis of cultured E14.5 tooth germs at 37, 33, 29, 25, and 4 °C for 0, 1, 3, 5, 7, and 10 days (n = 3). The mRNA expressions of *Epfn*, *AmeloD*, *Ambn,* and *Dspp* were normalized to *Gapdh*. Error bars represent the mean ± SD. (**e**) Immunofluorescence of cultured E14.5 tooth germs at 37, 25, and 4 °C for 10 days. Green: EPFN, blue: DAPI. Dashed lines represent the border of the dental epithelium and mesenchyme. *de* dental epithelium, *dm* dental mesenchyme. Scale bars, 100 µm. (**f**–**i**) RT-qPCR analysis of cultured E14.5 tooth germs at 25 and 4 °C for 10 days (n = 3). The mRNA expressions of *Sox2*, *Pitx2*, *Pax9*, and *Msx1* were normalized to *Gapdh*. *p < 0.05. Error bars represent the mean ± SD.

Since tooth morphogenesis was delayed by low-temperature culture, we next investigated gene expression changes in differentiation markers: *Epfn, AmeloD,* ameloblastin (*Ambn*), and dentin sialophosphoprotein (*Dspp*) (Fig. 5a–d). To examine these expression changes in cultured tooth germs, we cultured tooth germs at various temperatures and extracted total RNA on days 0, 1, 3, 5, 7, and 10 after culturing and performed RT-qPCR. The expression of differentiation markers *Epfn*, *AmeloD*, *Ambn*, and *Dspp* were increased in tooth germs cultured at 37 and 33 °C for 10 days, whereas the expression of these genes was not increased at 29, 25, and 4 °C (Fig. 5a–d). Tooth germs preserved at 4 °C demonstrated decreased expression of all these dental marker genes after 10 days of preservation (Fig. 5a–d).

We performed immunohistochemistry of the cultured tooth germs to examine the expression of EPFN (Fig. 5e). After 10 days of culture at 37 °C, the tooth germ showed late bell stage-like structures, whereas tooth germs cultured at 25 and 4 °C were retained at the cap stage. The expression of EPFN was only detected in the dental epithelium of the tooth germ cultured at 37 °C (Fig. 5e). These results suggest that low-temperature conditions delay morphogenesis as well as cellular differentiation in cultured tooth germs.

We observed that the long-term preservation at 25 °C demonstrated a higher success ratio than that at 4 °C (Fig. 2c). To clarify the difference between preservation at 25 °C and that at 4 °C, we performed RT-qPCR to investigate gene expression of dental epithelial stem cell marker, sex-determining region Y-box 2 (*Sox2*) and transcription factors expressed from the early development stage and involved in tooth development; paired like homeodomain 2*: Pitx2,* paired boxed 9*: Pax9*, and msh homeobox 1: *Msx1* of 10-day cultured tooth germs at 25 and 4 °C. Compared to tooth germs cultured at 25 °C, the expression of all of these genes was significantly downregulated in tooth germs cultured at 4 °C (Fig. 5f–i). These results suggest that tooth germs cultured at 25 °C could maintain the stemness of dental cells better than those at 4 °C, resulting in a higher recovery ratio after preservation.

### Subnormothermic temperature induces the expression of cold shock proteins

**Figure 6:**
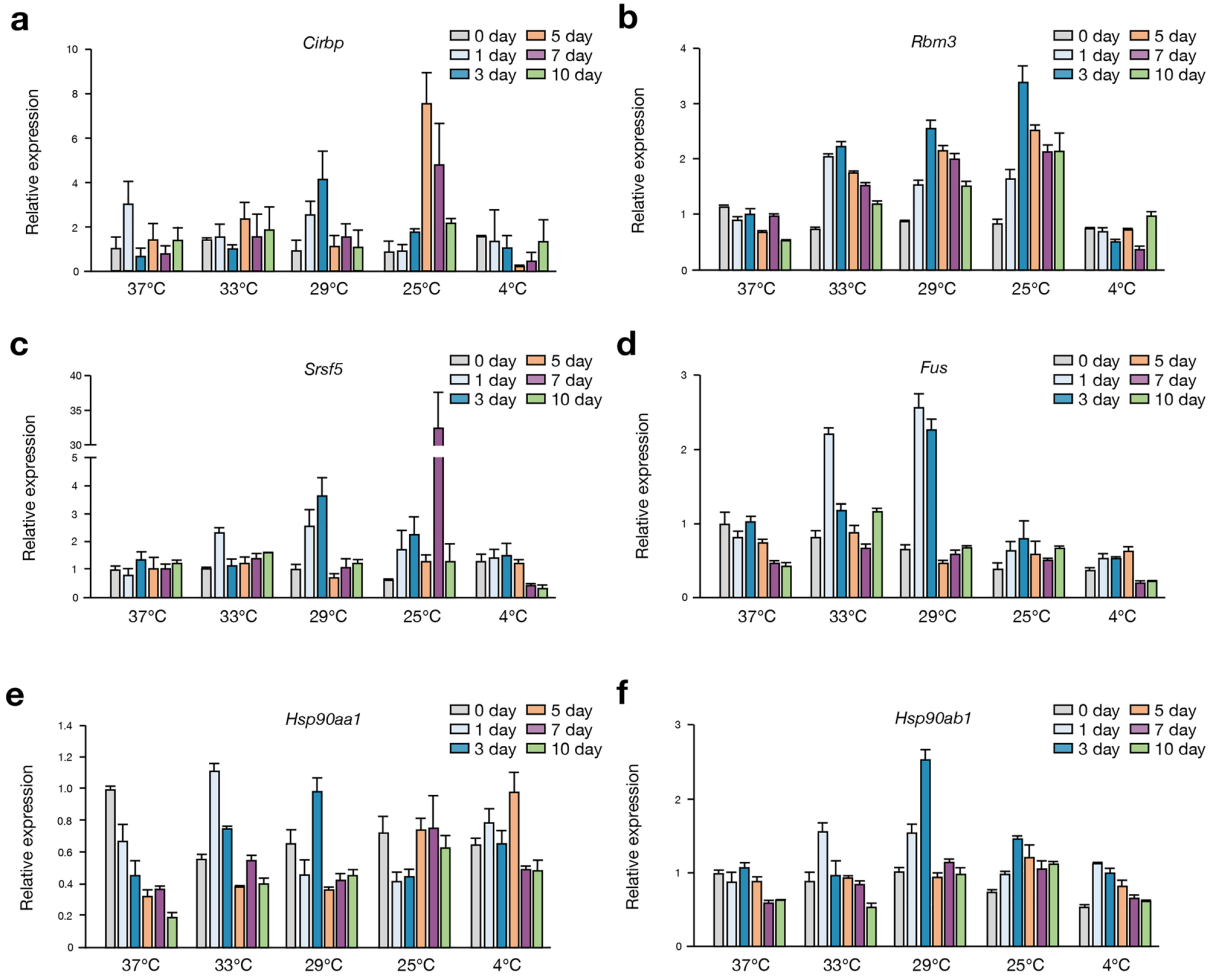
Subnormothermic temperature preservation upregulated the expression of cold shock protein genes. (**a**–**f**) RT-qPCR analysis of cultured E14.5 tooth germs at 37, 33, 29, 25, and 4 °C for 0, 1, 3, 5, 7, and 10 days (n = 3). mRNA expression of *Cirbp*, *Rbm3*, *Srsf5*, *Fus*, *Hsp90aa1,* and *Hsp90ab1* was normalized to *Gapdh*. Error bars represent the mean ± SD.

Several proteins whose expression is induced by low temperatures have been discovered, and these are called cold shock proteins (CSP). We performed RT-qPCR to investigate gene expression changes of the CSPs: cold-inducible RNA binding protein (*Cirbp*), RNA binding motif protein 3 (*Rbm3*), serine and arginine-rich splicing factor 5 (*Srsf5*), and transcripts regulated by Cirbp, fused in sarcoma (*Fus*), in cultured tooth germs. The expression of *Cirbp* was upregulated at 25 °C (Fig. 6a). The expression of *Rbm3* and *Srsf5* were gradually induced upon temperature changes from 33 to 25 °C (Fig. 6b,c). Similarly, the expression of *Fus* was upregulated at 33 and 29 °C (Fig. 6d). Notably, the expression of all CSP genes in tooth germs preserved at 4 °C revealed no significant change during 10 days; expression levels were similar to those cultured at 37 °C (Fig. 6a–d). We further performed RT-qPCR to investigate gene expression of heat shock proteins (HSP), heat shock protein 90, alpha, class A member 1 (*Hsp90aa1*) and heat shock protein 90 alpha, class B member 1 (*Hsp90ab1*). Although the expression of *Hsp90ab1* in 3-day cultured tooth germs at 29 °C was slightly upregulated (Fig. 6f), there was no change in the expression of *Hsp90aa1* and *Hsp90ab1*, regardless of culture temperature in general (Fig. 6e,f). These results suggest that subnormothermic temperatures induce the expression of CSP, suggesting that CSP may affect the subsequent recovery of organogenesis.

### Controlling CO_2_ is not necessary for tooth germ preservation in subnormothermic temperature culture systems

**Figure 7:**
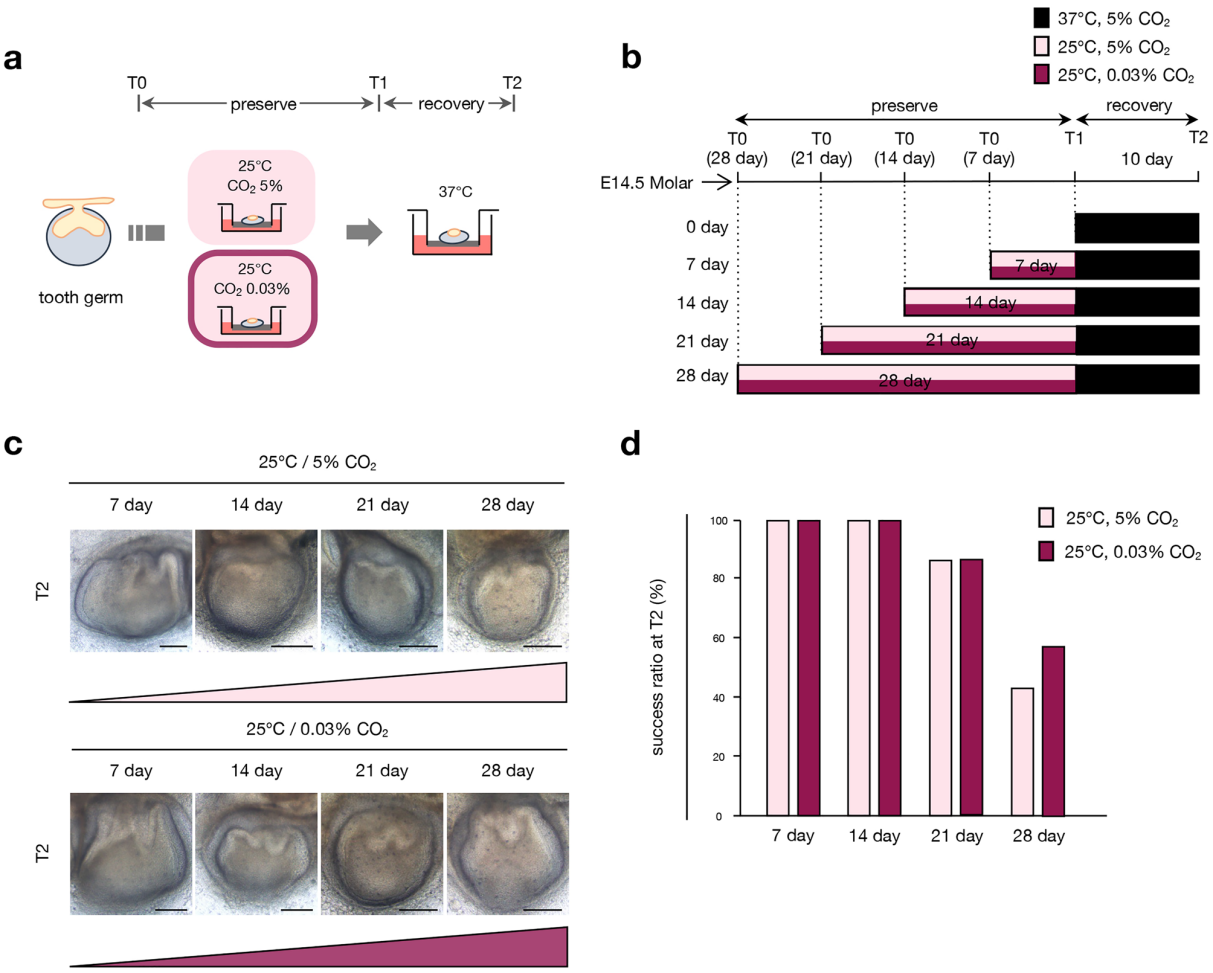
Concentration of CO_2_ does not affect the morphogenesis of cultured tooth germs. (**a**) A schematic of the experimental procedure for comparing conditions with different concentrations of CO_2_ using E14.5 tooth germ organ culture. T0: preservation temperature starting point, T1: preservation temperature endpoint, T2: recovery temperature endpoint. (**b**) Time course representation of tissue preservation in different concentrations of CO_2_ conditions (5% or 0.03%) for 7, 14, 21, and 28 days followed by recovery at 37 °C for 10 days. (**c**) A photographic analysis of cultured E14.5 tooth germs in conditions with different concentrations of CO_2_ is represented in (**b**). Scale bars, 200 µm. (**d**) Analysis of the development rate of cultured tooth germs under conditions with different concentrations of CO_2_ in (**c**) (n = 7).

In general, culturing cells and organs requires a CO_2_ incubator, which is adjusted to a 5% CO_2_ concentration, to stabilize the pH of the culture medium^40^. The temperature-dependent preservation indicates that 25 °C is the most suitable temperature for organ preservation. We next examined whether the concentration of CO_2_ affects the preservation of the tooth germ. We dissected E14.5 tooth germs and cultured them at different concentrations of CO_2_: control (5% CO_2_ concentration) and low-concentration groups (0.03% CO_2_ concentration, the same level as atmospheric CO_2_ concentration) for 7–28 days at 25 °C. After the preservation period, the tooth germs were recovered at 37 °C for 10 days in an atmosphere of 5% CO_2_ (Fig. 7a,b). Different concentrations of CO_2_ did not affect the development of tooth germs cultured at 25 °C for 28 days, and they resumed tooth germ morphogenesis at 37 °C (Fig. 7c,d). These results suggest that CO_2_ concentration may not affect organ preservation at subnormothermic temperatures.

## Discussion

In this study, we provided a simple model for screening the appropriate preservation temperature for regenerated tissues using a mouse organ culture system. This method may also be valuable for screening culture media or drugs for long-term storage in the next generation of regenerative medicine. In this study, we demonstrated that tissue development could be controlled by temperature and preservation time can be extended using an ex vivo culture system. Lower temperature delayed mouse tooth development in the ex vivo culture system and resumed development by the subsequent culture at 37 °C. Tooth germs preserved at 25 °C for 28 days exhibited recovery after preservation, suggesting that subnormothermic temperatures could help in the long-term preservation of tissues. Furthermore, tooth germs preserved with 0.03% CO_2_ showed similar recovery rates to those preserved with 5% CO_2_. These findings revealed the importance of the temperature-dependent preservation of tissues.

There are several possible reasons why subnormothermic temperature culture allows for the long-term preservation of cells and organs without subsequent damage. One of them is that culturing at hypothermic temperatures reduces the rate of cell metabolism and prevents cell damage due to oxidative stress^41^. We demonstrated that low temperatures delayed the morphogenesis and development of cultured mouse tooth germs. The developmental arrest of organ culture induced by low temperature is also reported using organ culture of the palatal mucosa^42^. Notably, morphogenesis and cellular differentiation were suppressed at low temperatures. The expression of *Ambn* and *Dspp*, markers of late differentiation, was induced in tooth germs cultured at 37 °C for 10 days, indicating that dental cells were differentiated using an ex vivo organ culture system at this temperature. While tooth germs cultured at 25 °C for 10 days did not exhibit induction of *Epfn*^43,44^ or early differentiation marker *AmeloD*^45,46^. These findings suggest that a temperature of 25 °C may arrest the differentiation of dental cells. In contrast, tooth germs cultured at 4 °C for 10 days showed a decrease in expression of dental epithelial stem cell marker, *Sox2,* compared to those at 25 °C, even though the expression of differentiation markers did not increase. This may be due to the loss of stemness of dental epithelial cells in tooth germs cultured at 4 °C.

Similarly, the expression of genes involved in early tooth development, *Pitx2*, *Pax9*, and *Msx1*, was decreased in tooth germs cultured at 4 °C. Notably, loss of genes like *Sox2*^47^, *Pitx2*^48,49^, *Pax9*^50,51^, and *Msx1*^52,53^ reportedly cause tooth agenesis and delayed tooth development in mice or humans, suggesting that long-term storage at 4 °C may be an unsuitable condition for maintaining expression of these genes. Collectively, 25 °C could be the optimal temperature to keep regenerated organs in good condition for transplantation. Several studies reported that CSP such as CIRBP, RBM3^54^, and SRSF5^55^ are induced by the hypothermic temperature. However, the function of CSP in organ development remains unclear. Furthermore, CIRBP and RBM facilitate cell proliferation and prevent apoptosis^56,57^. In this study, the expression of CSP genes, such as *Cirbp, Rbm3,* and *Srsf5*, did not change significantly in tooth germs preserved at 4 °C, but the expression of CSP increased in tooth germs cultured at subnormothermic temperatures. These data suggest that CSP may affect the maintenance of stem cells in developing tissues and allow for long-term preservation at subnormothermic temperatures. The role of CSP during subnormothermic temperature culture needs to be further analyzed by researchers. In this study, storage at − 80 °C was considered as a control for the subnormothermic culture model (Fig. 1a). However, this method follows the traditional technique of cell preservation in conventional cell culture. The progress in the cryopreservation of organs is remarkable, with the development of various solutions^12^.

Moreover, we examined the effect of CO_2_ concentration on tissue preservation as another factor essential for organ culture. The concentration of CO_2_ is vital to maintaining pH in the culture medium during organ culture, and it is mostly maintained at 5% in a CO_2_ incubator^40^. Notably, we observed no significant difference in the morphology of tooth germs cultured at under 5% CO_2_ and under 0.03% CO_2_ (equivalent to the CO_2_ concentration in air) at 25 °C. This finding suggests that the regulation of CO_2_ concentration may not be essential for tissue preservation when using the subnormothermic temperature culture method. This implies that if it is no longer necessary to culture tissues in a CO_2_ incubator, it may be possible to preserve and transport tissues using simpler systems. These findings may contribute to the development of strategies for the optimal preservation and transport of tissues in the future.

The present study suggests that it is possible to temporarily cease organ development and preserve tissues in the long-term using temperature-dependent culture. Notably, we have successfully preserved tissues for approximately 1 month using this temperature-dependent preservation method. However, we have performed experiments only on developing tissues formed by epithelial–mesenchymal interactions, such as teeth and submandibular glands, and further experiments are required to verify whether these phenomena can be applied to other organs. This is the limitation of this study. The conditions identified by our study are only suitable for preserving tooth germs at the stage of organogenesis, and cannot be applied to human organ transplantation, which involves fully developed organs. Appropriate storage conditions for larger tissues and effects on blood vessels must be considered for such processes. However, our findings are likely to provide useful insights for research on the conditions necessary for long-term preservation of organ transplantation and long-term preservation of regenerated tissues. Also, for human organ transplantation, preservation at lower temperatures and new cryopreservation methods have been developed, and our findings do not replace these methods. However, our finding can be effectively used for organ regeneration studies in small animals such as mice, and it may be effective for organ regeneration and preservation of regenerated organs such as minor salivary glands and hairs in humans.

**Figure 8:**
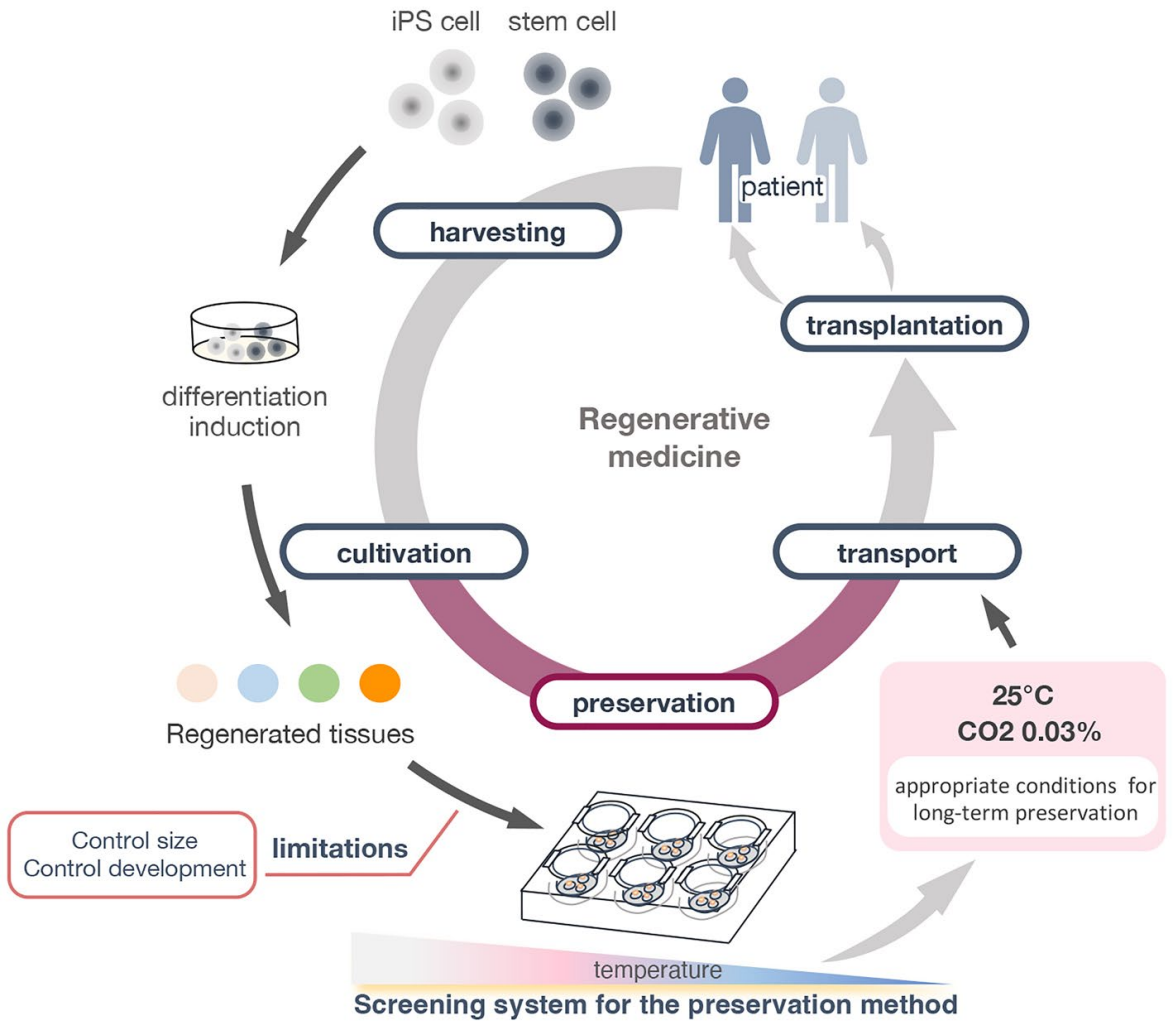
Summary diagram of the results of tissue preservation conditions analyzed in this study.

In this study, we have provided a roadmap for culturing and preserving tissues for use in regenerative organ transplantation technology which will be the focus of future studies (Fig. 8). Proper control of culture temperature allows the developmental stages of tissues to be regulated, resulting in the timely delivery of tissues to patients in need (Fig. 8). The results from this study are a step forward for such advancements in organ transplantation technology and regenerative medicine in small animals, and it may also be informative for the development of methods for transplanting organs into larger animals.

## Methods

### Organ cultures

The experimental animal protocol was approved by the Ethics Committee of the Kyushu University Animal Experiment Center (protocol number; A20-281-1). All procedures were performed in accordance with the relevant guidelines and regulations of the Kyushu University, and the study was performed in accordance with ARRIVE (Animal Research: Reporting of In Vivo experiments) guidelines. Pregnant mice were euthanized by medetomidine, midazolam, and butorphanol intraperitoneal administration and the embryos were dissected immediately. Tooth germs of mandibular molars were dissected from E14.5 mice embryos and placed on cell culture inserts (BD Falcon, BD Biosciences, Franklin Lakes, NJ, USA), and grown using an air–liquid interface culture technique in Dulbecco’s modified Eagle’s medium (DMEM)/F-12, supplemented with 20% fetal bovine serum (Gibco/Life Technologies, Waltham, MS, USA), 180 g/mL ascorbic acid, 2 mM l-glutamine, and 50 units/mL penicillin/streptomycin at 37 °C in a humidified atmosphere of 5% CO_2_, as described previously^31–34^. Submandibular glands were dissected from E13.5 mice embryos, placed on cell culture inserts, and grown in the same condition as tooth germs. To record the development of the cultured samples, images were captured daily under the microscope IX71 (Olympus, Tokyo, Japan) during the culture period. The development processes of cultured tooth germs were classified into five scores for evaluation: score 0, no noticeable change from the starting point; score 1, epithelium thickening; score 2, epithelial invagination into the mesenchyme; score 3, multiple cusp formation; score 4, final morphogenesis; score 5, differentiation. E13.5 submandibular glands were cultured for 2 days, and the size was determined using ImageJ software (Wayne Rasband, National Institutes of Health, Bethesda, MA, USA).

### Temperature and CO_2_ control for organ culture

In the low-temperature culture, the temperature was set at 25 and 4 °C, respectively. Tooth germs were cultured in an incubator at 25 °C in a humidified atmosphere of 5% or 0.03% CO_2_ for 7–28 days. Tooth germs were also cultured in a refrigerator at 4 °C with the same CO_2_ concentration (0.03%) as in the atmosphere. After low-temperature culture, tooth germs were cultured at 37 °C in a humidified atmosphere of 5% CO_2_ for 10 days. Submandibular glands were cultured at 25 or 4 °C for 7 days under the same conditions as the tooth germs and then cultured at 37 °C in a humidified atmosphere of 5% CO_2_ for 2 days. We changed to a fresh medium every 3 days and captured the images with the microscope IX71 (Olympus, Tokyo, Japan) daily for record-keeping.

### Immunohistochemistry

Cultured tooth germs were embedded in optimal cutting temperature (OCT) compound (4583; Sakura, Tokyo, Japan) at − 80 °C. Frozen sections were prepared by cutting them at a thickness of 10 µm using LEICA CM 1860 (Leica Biosystems, Wetzlar, Germany). Immunostaining of the frozen sections was performed using primary antibodies against EPFN raised as previously described^44^ (1:500) for 16 h at 4 °C. Tissue sections were then incubated with species-specific secondary antibodies conjugated with an Alexa 488 fluorescent dye (Life Technologies, Waltham, MS, USA) for 1 h at room temperature. Nuclei were visualized using DAPI staining. Sections were mounted with Vectashield mounting medium (Mountant, PermaFluor, TA-006-FM, Thermo Scientific, Waltham, MS, USA). Images were captured on a Zeiss LSM700 confocal laser scanning microscope (Carl Zeiss).

### RNA isolation and RT-qPCR analysis

Total RNA was isolated from cultured tooth germs dissected from E14.5 mice embryos using TRIzol reagent (Life Technologies, Waltham, MS, USA), then purified using an RNeasy Mini kit (Qiagen, Venlo, Netherlands). cDNA was synthesized using SuperScript III reverse transcriptase reagent (Life Technologies, Waltham, MS, USA). Specific forward and reverse primers were used for qRT-PCR. The primer sequences were as follows: *Epfn*, 5′-TGTTCTCCCTCTTTCCCCAC-3′ and 5′-GTGAGAGGTGGCTGGTTTTG-3′; *AmeloD*, 5′-ACTACGACGCCTACACTGGG-3′ and 5′-ATGAAGGCAGGCTCGAACGG-3′; *Ambn*, 5′-ACAACGCATGGCGTTTCCAA-3′ and 5′-ACCTTCACTGCGGAAGGATA-3′; *Dspp*, 5′-CATGAAACGACGCCTCAGAG-3′ and 5′-CATCCTCCTCTACCCCGTTC-3′; *Sox2*, 5′-GGCAATCAAATGTCCATT-3′ and 5′-TCCTTCCTTGTCTGTAAC-3′; *Pitx2*, 5′-CCGACTCCTCCGTACGTTTA-3′ and 5′-ATACTGGCAAGCACTCAGGT-3′; *Pax9*, 5′-GCTGCCCTACAACCACATTT-3′ and 5′-CTCACTCCTTGGTCGGTGAT-3′; *Msx1*, 5′-CTGCCCGAAACCCATGATC-3′ and 5′-CCGAGTGGCAAAGAAGTCAT-3′; *Cirbp*, 5′-CTACTATGCCAGCCGGAGTC-3′ and 5′-GGACACAAGGGTTCACCGAG-3′; *Rbm3*, 5′-CAGCAGCTTTGGGCCTATCT-3′ and 5′-GATCAACTCGGATTTGGCGC-3′; *Srsf5*, 5′-GTCCGGTAGGAAACACTAGCC-3′ and 5′-GATCCGTCCGTAACCCTTGAA-3′; *Fus*, 5′-AGCTCCCCAGGGATATGGTT-3′ and 5′-GCTCTGAGAACTGCCACCAT-3′; *Hsp90aa1*, 5′-ATCTGCTTCTGGGGACGAGA-3′ and 5′-CTGGTCCTTGGTCTCACCTG-3′; *Hsp90ab1*, 5′-GACCTGCCCCTGAACATCTC-3′ and 5′-GGCGTCGGTTAGTGGAATCT-3′; and glyceraldehyde 3-phosphate dehydrogenase (*Gapdh*), 5′-GGAGCGAGACCCCACTAACATC-3′ and 5′-CTCGTGGTTCACACCCATCAC-3′.

The expression level of each gene was normalized to that of *Gapdh*. qRT-PCR was performed using iQ SYBR Green Supermix (Bio-Rad, Hercules, CA, USA) with a CFX Connect Real-Time PCR detection system (Bio-Rad, Hercules, CA, USA).

### Statistics

All experiments were repeated at least three times to confirm reproducibility. Statistical significance was determined using a two-tailed unpaired Student’s *t*-test with Prism 9 (GraphPad Software). One-way ANOVA was utilized for quantification between multiple groups. Differences with p values < 0.05 were considered statistically significant.

## Supplementary Information

Supplementary Figure S1.

## Data Availability

All data generated or analyzed during this study are included in this published article (and its Supplementary Information files).
