# Supplementary material for: Development of a novel ex vivo organ culture system to improve preservation methods of regenerative tissues
Source: Sci Rep. 2023 Feb 27;13:3354. doi: 10.1038/s41598-023-29629-2 (PMC9971270; doi:10.1038/s41598-023-29629-2)
Supplement: Supplementary file 1 — Supplementary Figure S1. [file 41598_2023_29629_MOESM1_ESM.pdf]

Supplemental information

Development of a novel ex vivo organ culture system to improve preservation methods of regenerative tissues

Tomomi Yuta, Tian Tian, Yuta Chiba, Kanako Miyazaki, Keita Funada, Kanji Mizuta, Yao Fu, Jumpei Kawahara, Tsutomu Iwamoto, Ichiro Takahashi, Satoshi Fukumoto, Keigo Yoshizaki

Supplemental Figure S1

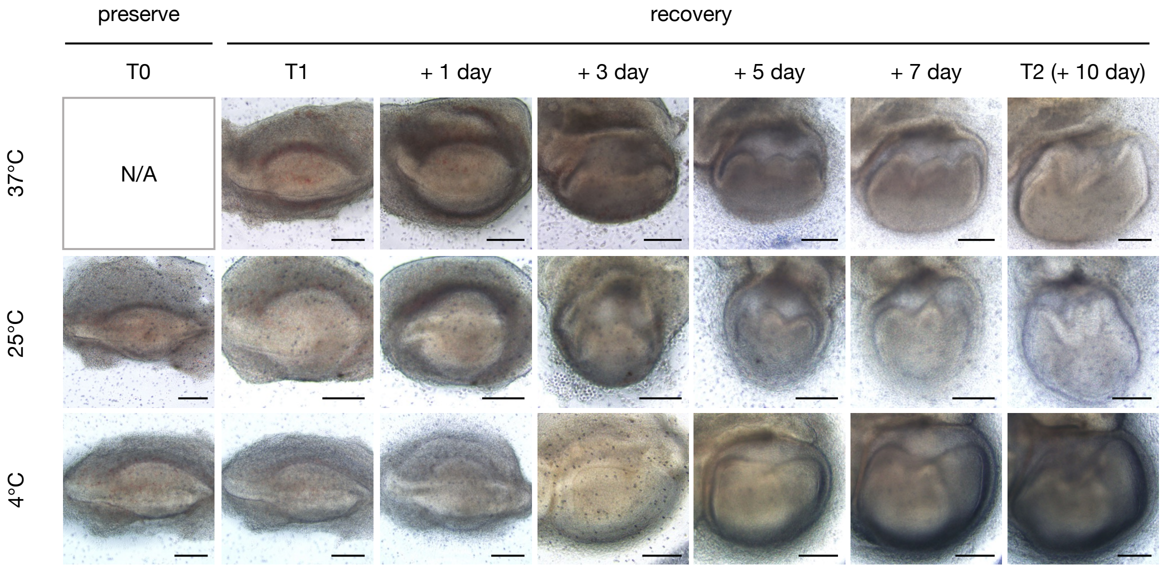

Supplementary Figure 1. Photographic analysis of cultured E14.5 tooth germs in low-temperature preservation conditions related to Fig. 1c. Scale bars, 200 µm.
